# Ribonucleotide reductase subunits M1 and M2 mRNA expression levels and clinical outcome of lung adenocarcinoma patients treated with docetaxel/gemcitabine

**DOI:** 10.1038/sj.bjc.6604344

**Published:** 2008-04-15

**Authors:** J Souglakos, I Boukovinas, M Taron, P Mendez, D Mavroudis, M Tripaki, D Hatzidaki, A Koutsopoulos, E Stathopoulos, V Georgoulias, R Rosell

**Affiliations:** 1 Laboratory of Tumor Cell Biology, School of Medicine, University of Crete, Crete, Greece; 2 Department of Medical Oncology, University General Hospital of Heraklion, Crete, Greece; 3 Second Department of Medical Oncology, ‘Theagenion’ Cancer Hospital of Thessaloniki, Thessaloniki, Greece; 4 Catalan Institute of Oncology, Medical Oncology Service, Hospital Germans Trias i Pujol, Badalona (Barcelona), Spain; 5 Department of Pathology, University General Hospital of Heraklion, Crete, Greece

**Keywords:** RRM1, RRM2, lung adenocarcinoma, docetaxel, gemcitabine, pharmacogenomics

## Abstract

Ribonucleotide reductase subunits M1 (RRM1) and M2 (RRM2) are involved in the metabolism of gemcitabine (2′,2′-difluorodeoxycytidine), which is used for the treatment of nonsmall cell lung cancer. The mRNA expression of RRM1 and RRM2 in tumours from lung adenocarcinoma patients treated with docetaxel/gemcitabine was assessed and the results correlated with clinical outcome. RMM1 and RMM2 mRNA levels were determined by quantitative real-time PCR in primary tumours of previously untreated patients with advanced lung adenocarcinoma who were subsequently treated with docetaxel/gemcitabine. Amplification was successful in 42 (79%) of 53 enrolled patients. Low levels of RRM2 mRNA were associated with response to treatment (*P*< 0.001). Patients with the lowest expression levels of RRM1 had a significantly longer time to progression (*P*=0.044) and overall survival (*P*=0.02) than patients with the highest levels. Patients with low levels of both RRM1 and RRM2 had a significantly higher response rate (60 *vs* 14.2%; *P*=0.049), time to progression (9.9 *vs* 2.3 months; *P*=0.003) and overall survival (15.4 *vs* 3.6; *P*=0.031) than patients with high levels of both RRM1 and RRM2. Ribonucleotide reductase subunit M1 and RRM2 mRNA expression in lung adenocarcinoma tumours is associated with clinical outcome to docetaxel/gemcitabine. Prospective studies are warranted to evaluate the role of these markers in tailoring chemotherapy.

Platinum-based chemotherapy is the standard treatment for patients with inoperable locally advanced and metastatic nonsmall cell lung cancer (NSCLC) as it prolongs survival and improves quality of life (Souquet *et al*, 1993). However, randomised studies have reported no substantial difference in terms of efficacy between various platinum-based regimens using new anticancer drugs (Schiller *et al*, 2002). Moreover, the toxicity profile of cisplatin can influence the patients' quality of life. Several randomised phase III studies have demonstrated that nonplatinum-containing regimens have substantial efficacy against advanced/metastatic NSCLC with a more favourable toxicity profile than the corresponding cisplatin-based regimens (Douillard *et al*, 2001; Georgoulias *et al*, 2001, 2005; Kosmidis *et al*, 2002).

Gemcitabine (2′,2′-difluorodeoxycytidine) is a deoxycytidine analogue that is incorporated into DNA and competitively inhibits DNA synthesis (Chabner, 1996). Ribonucleotide reductase (RR) is an enzyme of central importance in DNA synthesis (Cory and Sato, 1983). Ribonucleotide reductase catalyses the conversion of ribonucleotide 5′-diphosphates to their 2′-deoxynucleotide forms, a rate-limiting step in the production of 2′-deoxyribonucleoside 5′-triphosphates (dNTP) required for DNA synthesis (Cory, 1997). The RR holoenzyme consists of M1 (RRM1) and M2 (RRM2) subunits, and the holoenzymatic activity is modulated by levels of the M2 subunit (RRM2) (Tanaka *et al*, 2000).

RRM1, localised in 11p15.5, also acts as a putative tumour suppressor gene (Pitterle *et al*, 1999). 11p15.5, also known as LOH11A, is frequently lost in NSCLC, and loss of heterozygosity in this region has been correlated with poor survival in resected NSCLC patients (Bepler *et al*, 2002). However, RRM1 overexpression was related to gemcitabine resistance in human oropharyngeal epidermoid carcinoma KB cells (Goan *et al*, 1999). Ribonucleotide reductase subunit M1 mRNA expression by quantitative PCR significantly influenced time to progression and survival in stage IV NSCLC patients treated with gemcitabine/cisplatin (Rosell *et al*, 2003). Two subsequent studies confirmed that RRM1 mRNA expression levels were able to identify stage IV NSCLC patients likely to have good or poor survival when treated with gemcitabine/cisplatin (Rosell *et al*, 2004; Ceppi *et al*, 2006).

Ribonucleotide reductase subunit M2 itself is a dimer of two 44 kDa moieties, each containing a tyrosine free radical and nonhaeme iron (Thelander *et al*, 1985). Cells overexpressing RRM2 exhibit enhanced cellular invasiveness (Zhou *et al*, 1998), through activation of nuclear factor *κ*B (NF-*κ*B), which increases MMP-9 expression (Duxbury and Whang, 2007). There is limited information concerning the effect of tumoral RRM2 expression and response to gemcitabine in human tumours.

In a randomised multicentre trial comparing docetaxel/cisplatin and docetaxel/gemcitabine as front-line chemotherapy in NSCLC patients, a significantly higher objective response rate was achieved with docetaxel/gemcitabine in adenocarcinoma than in nonadenocarcinoma patients (Georgoulias *et al*, 2001). To further investigate this issue, we have carried out a multicentre phase II study to evaluate the impact of RRM1 and RRM2 mRNA expression in the tumours of lung adenocarcinoma patients treated with docetaxel/gemcitabine.

## MATERIALS AND METHODS

### Patients

Chemotherapy-naive patients with inoperable, histologically confirmed stage IIIB and IV adenocarcinoma of the lung and Eastern Cooperative Oncology Group performance status (PS) 0–2 were enrolled. Other eligibility criteria were the same as those reported in previous studies (Georgoulias *et al*, 2001). The study was approved by the ethics and scientific committees of the participating hospitals and was conducted according to the Declaration of Helsinki. All patients gave their signed informed consent prior to study entry. Gemcitabine (Gemzar; Eli Lilly, Indianapolis, IN, USA) (1000 mg m^−2^ on days 1 and 8) and docetaxel (Taxotere; Sanofi-Aventis, Collegeville, NJ, USA) (100 mg m^−2^ on day 8) with human granulocyte colony-stimulating factor support were administered every 3 weeks as previously described (Georgoulias *et al*, 2001), with dose adjustment if needed due to haematological and nonhaematological toxicity (Georgoulias *et al*, 2001). Patients were evaluated at baseline and before each third cycle of chemotherapy (Georgoulias *et al*, 2001).

### RRM1 and RRM2 assessment

All paraffin-embedded tumours were reviewed by an independent pathologist to ensure the validity of the specimen and define the most appropriate tumour area for microdissection. From each paraffin block of tumour, serial sections with a thickness of 5 *μ*m were prepared and then stained with nuclear Fast Red (Sigma-Aldrich, St Louis, MO, USA).

RRM1 and RRM2 gene expression analysis was performed in RNA isolated from tumour tissue specimens after laser capture microdissection (Palm, Oberlensheim, Germany), according to a proprietary procedure (patent pending EP05077417.3) of Pangaea Biotech, SA.

The RT-PCR was carried out with the addition of 2.5 *μ*l of template cDNA to 6.25 *μ*l Taqman Universal Master Mix (AB; Applied Biosystems, Foster City, CA, USA) with the addition of specific primers and probe for each gene and adjusted with diethylpyrocarbonate-treated water in a final volume of 12.5 *μ*l per reaction. The primer and probe sets were designed using Primer Express 2.0 Software (AB). Quantification of gene expression was performed using the ABI Prism 7900HT Sequence Detection System (AB). Primers and probe were designed according to the Ref Seq L10342 for RRM1 and NM_001034 for RRM2 (http://www.ncbi.nlm.nih.gov/LocusLink). The primers and 5′-labelled fluorescent reporter dye (6FAM) probe were as follows: RRM1: forward 5′-ACTAAGCACCCTGACTATGCTATCC-3′, reverse 5′-CTTCCATCACATCACTGAACACTTT-3′, probe 5′-CAGCCAGGATCGCTGTCTCTAACTTGCA-3′; RRM2: forward 5′-CCTGGCCAGCAAGACCG-3′, reverse 5′-TAGTTTTCGGCTCCGTGGG-3′, probe 5′-CGAGGAGGATCTTCCAGGA-3′; *β*-actin: forward 5′-TGAGCGCGGCTACAGCTT-3′, reverse 5′-TCCTTAATGTCACGCACGATTT-3′, probe 5′-ACCACCACGGCCGAGCGG-3′.

Relative gene-expression quantification was performed according to the comparative *C*_t_ method using *β*-actin as an endogenous control and commercial RNA controls (Stratagene, La Jolla, CA, USA) as calibrators. Final results were determined as follows: 2^−(Δ*C*t sample−Δ*C*t calibrator)^, where Δ*C*_t_ values of the calibrator and sample are determined by subtracting the *C*_t_ value of the target gene from the value of the housekeeping gene, *β*-actin. In all experiments, only triplicates with a standard deviation (s.d.) of the *C*t value <0.20 were accepted. In addition, genomic DNA contamination was excluded by nonreverse transcript RNA for each sample analysed.

### Study design

The trial was designed as a prospective phase II trial focused on biomarker analysis in lung adenocarcinoma patients treated with docetaxel/gemcitabine. The primary end point was the overall response rate. The study followed the optimal Simon two-step design. If a minimum objective response rate exceeding 35% was observed in the first 16 patients, 39 additional patients were enrolled in a 2-year (December 2001 to December 2003) time period (*α*=0.05, power 80%). All efficacy and toxicity results were assessed for all enrolled patients on an intent-to-treat basis. Median time to progression and overall survival were calculated from the start of treatment to the first documented disease progression or death, respectively. Quantitative PCR analyses yielded values that were expressed as ratios between two absolute measurements (gene of interest: internal reference gene). Cutoff points were calculated according to median value for the mRNA expression of each gene. Samples with mRNA expression above or equal to the median were considered as samples with high expression, whereas those with value below the median as samples with low expression. In addition, the gene mRNA expression levels were divided in quartiles (Q1 (lowest levels)–Q4 (highest levels)). Patients in the middle quartiles were considered as one group (Q2+Q3), and comparisons were performed among all three groups (Q1, Q2+3, Q4).

### Statistical analyses

The Mann–Whitney *t*-test was used to test a significant association between the continuous variable of gene expression and the dichotomous variables. Pearson's exact test was used to evaluate the correlation between RRM1 and RRM2 mRNA expression. The *χ*^2^ test was used for the association between gene expression and response. Binary logistic regression was carried out to evaluate which of the significant factors in the *χ*^2^ analysis had a significant influence on response. Cox's proportional hazards multivariate analysis was used to evaluate which of the significant factors at the univariate analysis had a significant influence on time to progression and overall survival. Logistic regression analysis was used to evaluate which of the significant factors at the univariate analysis had a significant influence on response. Statistical significance was set at *P*=0.05.

## RESULTS

### Patient characteristics

**Figure 1:**
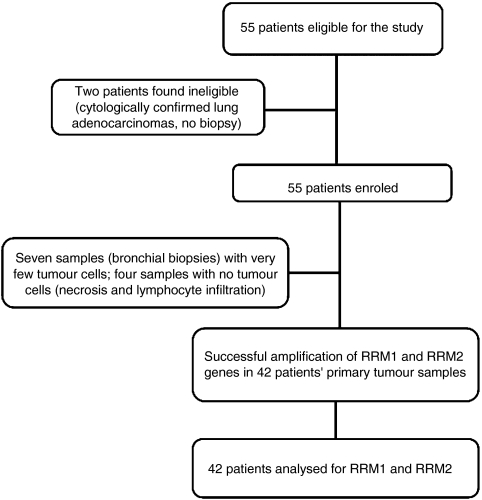
Flow chart showing patient progress through the study.

A total of 53 patients were enrolled. Patient characteristics are shown in Table 1. Forty-two (79%) samples were successfully amplified; the remaining 11 samples were not quantifiable because of insufficient tumour tissue or large amounts of necrosis in the tumour sample (Figure 1).

### Clinical outcome

In an intent-to-treat analysis, complete response was observed in four (7.5%) and partial response in 15 (28.3%) patients (overall response rate 35.8; 95% confidence interval: 19.6–46.9%). Fifteen (28.3%) patients had stable disease and 20 (37.7%) had progressive disease. There was no correlation between response and known clinical parameters (age, PS, disease stage or the number of involved sites). After a median follow-up period of 7.5 months (range, 0.5–46.2), the median time to progression was 4.3 months (range, 0.2–34.9), and the median overall survival was 10.1 months (range, 0.5–46.2); the 1-year survival rate was 40.2%. For the 42 patients evaluable for RRM1 and RRM2 mRNA expression, overall response rate was 30%, time to progression was 3.9 months and median overall survival was 9.8 months.

### RRM1 and RRM2 mRNA expression and clinical outcome

Tumour RRM1 and RRM2 mRNA expression levels ranged from 0.1 to 8.9 (median 1.04; mean 1.8±s.d. 1.15) and from 0.97 to 114.3 (median 8.83; mean 17.69±s.d. 20.56), respectively. There was no correlation between age, gender, PS or disease stage and RRM1 or RRM2 mRNA levels. Using the median expression levels as cutoff values, high (above or equal to the median) RRM1 mRNA expression was observed in 25 (59.5%) and low (below the median) in 17 (40.5%) patients, and high RRM2 mRNA expression in 22 (52.4%) and low in 20 (47.6%) patients. When patients were divided into quartiles according to their expression levels of RRM1, 11 patients were in the lowest quartile (0.1–0.36), 20 patients were in the intermediate quartiles (0.36–2.11) and 11 patients were in the highest quartile (2.11–8.9). When patients were divided into quartiles according to their expression levels of RRM2, 11 patients were in the lowest quartile (0.97–4.03), 20 patients in the intermediate quartiles (4.03–21.75) and 11 in the highest quartile (21.75–114.3) (Table 2).

When patients were divided into those with low *vs* those with high RRM1 and RRM2 expression, no correlation was found between RRM1 expression and clinical outcome. However, the 20 patients with low RRM2 expression attained a significantly higher response rate than the 22 patients with high RRM2 expression (54.5 *vs* 9%; *P*=0.002); there was no significant correlation between RRM2 expression and time to progression or survival (Table 2). When patients were divided by quartiles of RRM1 and RRM2 expression, the 11 patients in the lowest quartile of RRM1 expression had a longer time to progression (7.1 *vs* 1.7 months; *P*=0.04) and overall survival (10.6 *vs* 1.6 months; *P*=0.02) than the 11 patients in the highest quartile; in addition, there was a nonsignificant trend towards a higher response rate for patients in the lowest quartile, compared to those in the highest quartile (45.5 *vs* 20%; *P*=0.062) (Table 2). The 11 patients in the lowest quartile of RMM2 expression attained a significantly higher response rate than the 11 patients in the highest quartile (45.5 *vs* 0%; *P*=0.016), but there was no significant difference in time to progression or overall survival according to RRM2 expression quartiles (Table 2).

**Figure 2:**
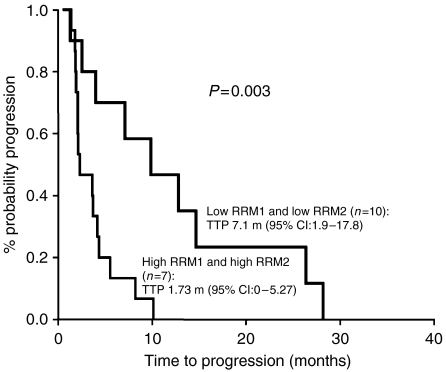
Time to progression according to low *vs* high levels of both RRM1 and RRM2.

**Figure 3:**
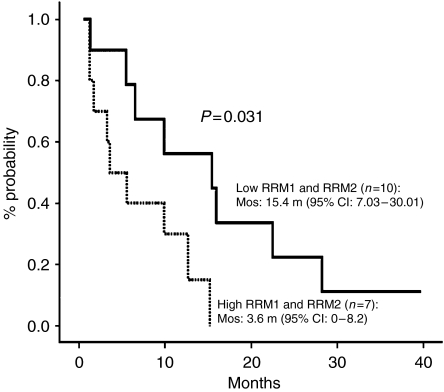
Overall survival according to low *vs* high levels of both RRM1 and RRM2.

When patients were classified according to their expression levels of both RRM1 and RRM2, the 10 patients with low levels of both RRM1 and RRM2 attained a higher response rate, time to progression and overall survival than the seven patients with high levels of both genes (response rate: 60 *vs* 14.2%, *P*=0.049; time to progression: 9.9 *vs* 2.3 months, *P*=0.003 (Figure 2); overall survival: 15.4 *vs* 3.6 months, *P*=0.031 (Figure 3)) (Table 2). No other significant correlation was observed between clinical outcome and the combined expression levels of RRM1 and RRM2 mRNA (Table 2).

### Univariate and multivariate analyses

The univariate analysis (Table 3) showed that RRM2 mRNA expression was significantly associated with response rate (*P*<0.001). The multivariate analysis showed that high expression of RRM2 – but not of RRM1 – was significantly associated with poor response (odds ratio: 31.5; *P*=0.002) (Table 3). In the univariate and multivariate analyses of survival, only PS emerged as a significant prognostic factor for survival (hazard ratio: 2.26; *P*=0.024) (Table 4).

## DISCUSSION

The present study has demonstrated for the first time a positive correlation between tumour mRNA expression of RRM2 and response to a gemcitabine-based combination in patients with lung adenocarcinomas. Low RRM2 mRNA expression was associated with a significantly higher response rate compared to that of patients with high RRM2 mRNA expression. In fact, only two (9%) of 22 patients with high RRM2 expression responded to docetaxel/gemcitabine. Furthermore, the multivariate analysis revealed that RRM2 expression was an independent marker for response (*P*=0.002). The probability of response in patients with low RRM2 expression was 31.5 times higher than that of patients with high RRM2 expression. In addition, patients with RRM1 mRNA expression in the lowest quartile attained a significantly higher time to progression (*P*=0.044) and overall survival (*P*=0.02) and showed a trend towards a higher response rate (*P*=0.06), compared to patients in the highest quartile of RRM1 expression. Furthermore, patients with low expression of both RRM1 and RRM2 derived the maximum benefit from docetaxel/gemcitabine, with a significantly higher response rate (*P*=0.049), time to progression (*P*=0.003) and overall survival (*P*=0.031). Despite the fact that caution is needed when interpreting these results, due to the small number of patients evaluated, our data suggest that the molecular profile of the primary tumour could be used as a marker for response and clinical outcome of patients with lung adenocarcinomas treated with docetaxel/gemcitabine.

The results of the present study are along the lines of several other reports demonstrating that tumour mRNA expression of RRM1 was correlated with clinical response to gemcitabine/cisplatin in stage IV NSCLC (Rosell *et al*, 2003, 2004; Ceppi *et al*, 2006). In addition, inhibition of RRM2 mRNA expression *in vitro* with the use of interference RNA enhanced chemosensitivity of pancreatic adenocarcinomas to gemcitabine (Duxbury *et al*, 2003, 2004). Apart from the well-known role of RRM2 in maintaining DNA integrity, several studies have reported that RRM2 has additional functions that influence the invasive phenotype. Increased RRM1 and RRM2 activity was identified in highly metastatic tumour cells (Schallreuter *et al*, 1992), whereas overexpression of RRM2 in human oral carcinoma cells was shown to be associated with increased invasive potential (Zhou *et al*, 1998), probably through NF-*κ*B and MMP-9 (Duxbury and Whang, 2007).

In conclusion, the results of the present study revealed that the efficacy of docetaxel/gemcitabine in lung adenocarcinoma patients is associated with RRM1 and RRM2 mRNA expression, thus indicating the importance of tailoring treatment. As this study was conducted in patients with lung adenocarcinomas, it is unclear whether these observations are also valid in patients with squamous cell carcinomas, and the sample size in the present study requires the results to be interpreted with caution. The role of RRM1 and RRM2 should be further investigated in an adequately statistically powered, independent set of samples. In addition, prospective studies using gemcitabine, either as a single agent or in combination with other drugs, are warranted to further clarify the relation between RRM1 and RRM2 coexpression and tumour chemosensitivity. The potential predictive role of RRM1 and RRM2 mRNA expression warrants examination in malignancies where gemcitabine-based therapy is standard, such as pancreatic cancer. Finally, prospective studies are warranted to clarify the importance of RRM1 and RRM2 expression for tailoring treatment of patients with NSCLC.

## Figures and Tables

**Table 1:** Patient characteristics

	**Patients**	
	**Number**	**%**
*Gender*		
Male	45	85
Female	8	15
		
*Age (years)*		
Median	60	
Range	39–75	
		
*Performance status (ECOG)*		
0	35	66
1	16	30
2	2	4
		
*Stage*		
IIIB	12	23
IV	41	77
		
*Number of organs involved*		
1	16	30
2	20	38
⩾3	17	32
		
*RRM1 mRNA expression*		
Samples analysed	53	100
Samples successfully amplified	42	79
Missing values	11	21
		
*RRM2 mRNA expression*		
Samples analysed	53	100
Samples successfully amplified	42	79
Missing values	11	21

Abbreviations: ECOG=Eastern Cooperative Oncology Group; RRM1=ribonucleotide reductase subunit M1; RRM2=ribonucleotide reductase subunit M2.

**Table 2:** Tumour RRM1 and RRM2 mRNA expression and clinical outcome

			**Time to progression (months)**		**Overall survival (months)**		**Response *N* (%)**		
		***N* (%)**	**Median (95% CI)**	***P***	**Median (95% CI)**	***P***	**CR+PR**	**SD+PD**	***P***
High *vs* low	Low RRM1	17 (40.5)	4.0 (3.1–5.8)	0.43	9.8 (4.2–15.4)	0.31	7 (41)	10 (59)	0.23
	High RRM1	25 (59.5)	3.7 (1.9–5.4)		5.5 (0.7–10.3)		6 (24)	19 (76)	
	Low RRM2	20 (47.6)	3.6		6.5		11 (54.5)	9 (64.5)	0.002
	High RRM2	22 (52.4)	4.9	0.38	6.8	0.9	2 (9)	19 (91)	
Quartiles	RRM1 by quartiles								
	Q1 (low)	11	7.1(1.8–12.4)	0.04^a^	10.6 (5.6–15.5)	0.02^a^	5 (45.5)	6 (54.5)	0.06^a^
	Q2+Q3	20	4.0 (0.8–7.2)	NS^b^	5.5 (3.8–12.6)	NS^b^	6 (30)	70	NS^b^
	Q4 (high)	11	1.73 (0.6–2.0)		1.6 (1.4–1.8)		2 (20)	80	
	RRM2 by quartiles								
	Q1 (low)	11	5 (1.6–9.8)	0.18^a^	10.6 (4.3–19.7)	0.14^a^	5 (45.5)	6 (54.5)	0.016^a^
	Q2+Q3	20	3.6 (1.1–6.1)	NS^b^	9.5 (1.4–16.1)	NS^b^	8 (40)	12 (60)	0.035^c^
	Q4 (high)	11	3.6 (0.1–5.6)		5.5 (0.5–10.5)		0 (0)	11 (100)	
RRM1 and RRM2	Low RRM1 and low RRM2	10	9.9 (1.9–17.8)	0.003^d^	15.4 (7.0–30.1)	0.03^d^	6 (60)	4 (40)	0.049^d^
	High RRM1 and high RRM2	7	2.3 (0–5.3)		3.6 (0–8.2)		1 (14.2)	6 (85.8)	
	Low RRM1 and high RRM2	15	3.6 (0.32–6.8)	0.1^e^	6.4 (4.4–8.3)	0.17^e^	3 (20)	12 (80)	0.05^e^
	High RRM1 and low RRM2	10	3.8 (0–7.2)	0.4^e^	6.9 (0.1–9.3)	0.15^e^	3 (30)	7 (70)	0.06^e^
									

Abbreviations: CI=confidence interval; CR=complete response; PD=progressive disease; PR=partial response; SD=stable disease; RRM1=ribonucleotide reductase subunit M1; RRM2=ribonucleotide reductase subunit M2.

a *P*-value: Q1 *vs* Q4.

b *P*-value: Q1 *vs* Q2+3 and Q2+3 *vs* Q4.

c *P*-value: Q2+3 *vs* Q4.

d *P*-value: both low *vs* both high.

e Both low *vs* RRM1 low/RRM2 high or RRM1 high/RRM2 low.

**Table 3:** Univariate and multivariate analyses for response

	**Odds ratio**	**95% CI**	***P*-value**
*Univariate analysis*			
Age (⩽65 *vs* >65 years)	1.01	0.69–1.34	0.97
Gender (male *vs* female)	1.23	0.7–1.83	0.64
Performance status (0 *vs* 1–2)	1.54	0.87–2.04	0.15
Stage (III *vs* IV)	1.12	0.67–1.41	0.42
RRM1 mRNA (low *vs* high)	1.38	0.67–2.91	0.31
RRM2 mRNA (high *vs* low)	37.5	3.8–369.8	<0.001
			
*Multivariate analysis*			
RRM2 mRNA (high *vs* low)	31.5	3.5–283.3	0.002

Abbreviations: CI=confidence interval; RRM1=ribonucleotide reductase subunit M1; RRM2=ribonucleotide reductase subunit M2.

**Table 4:** Univariate and multivariate analyses for survival

	**Hazard ratio**	**95% CI**	***P*-value**
*Univariate analysis*			
Age (⩽65 *vs* >65 years)	1.04	0.45–2.41	0.912
Gender (male *vs* female)	1.82	0.73–4.49	0.192
Performance status (0 *vs* 1–2)	2.64	1.32–4.19	0.017
Stage (III *vs* IV)	1.46	0.57–3.75	0.421
RRM1 mRNA (low *vs* high)	1.58	0.78–3.20	0.198
RRM2 mRNA (high *vs* low)	1.85	0.75–4.56	0.178
			
*Multivariate analysis*			
Performance status (0 *vs* 1–2)	2.26	1.11–4.59	0.024

Abbreviations: RRM1=ribonucleotide reductase subunit M1; RRM2=ribonucleotide reductase subunit M2.
